# MicroRNA 876-5p modulates EV-A71 replication through downregulation of host antiviral factors

**DOI:** 10.1186/s12985-020-1284-8

**Published:** 2020-02-05

**Authors:** Peng Xu, Hwa Xu, Hsu Sheng Cheng, Han-Hsiang Chan, Robert Y. L. Wang

**Affiliations:** 1 Xiangyang No.1 People’s Hospital and Hubei University of Medicine, Xiangyang, Hubei Province China; 2 College of Resources and Environment Qingdao Agricultural Unviersity, Qingdao, China; 3 Graduate Institute of Biomedical Sciences, College of Medicine, Chang Gung University, Taoyuan, 33302 Taiwan; 4 Department of Biomedical Sciences, College of Medicine, Chang Gung University, Tao-Yuan, 33302 Taiwan; 5 Division of Pediatric Infectious Diseases, Department of Pediatrics, Chang Gung Memorial and Children’s Hospital, Linkou, 33305 Taiwan

**Keywords:** Enterovirus A71, microRNA-876-5p, CREB5, Antiviral protein

## Abstract

**Background:**

Human enterovirus 71 (EV-A71) is a non-enveloped virus that has a single stranded positive sense RNA genome. In a previous study, we showed that miR-876-5p upregulation was observed in the serum of patients with severe EV-A71 infection. Micro-876-5p (miR-876-5p) is a circulating miRNA that can be identified to modulate EV-A71 infections through both in vitro and in vivo studies. However, the regulatory mechanisms that involve miR-876-5p in the EV-A71 infection cycle remain unclear.

**Methods:**

We demonstrated that miR-876-5p facilitated EV-A71 replication and expression by overexpression and knocking-down of miR-876-5p through the transfection of miR-876-5p plasmid and miR-876-5p inhibitor. Although miR-876-5p suppressed CREB5 expression, luciferase reporter assay confirmed this. We also evaluated the role of miR-876-5p in the EV-A71 infection cycle by CREB5 mediated by transfection with an anti-miR-876-5P inhibitor or in combination with an si-CREB5 plasmid.

**Results:**

MicroR-876-5p was upregulated in EV-A71-infected neuroblastoma cells. Overexpression of miR-876-5p or knockdown of cyclic-AMP responsive element binding protein 5 (CREB5) promoted EV-A71 replication. The downregulation of miR-876-5p inhibited the accumulation of viral RNA and the production of viral proteins. Interestingly, CREB5 overexpression also suppressed EV-A71 replication. Our in vitro studies reveal that miR-876-5p directly targets CREB5. Finally, downregulation of CREB5 protein abated the inhibitory effect of anti-miR-876-5p and induced inhibitory effect of EV-A71 replication.

**Conclusions:**

Our results suggest that intracellular miR-876-5p promotes EV-A71 replication indirectly by targeting the host CREB5 protein.

## Background

Human enterovirus A71 (EV-A71) is a single-stranded positive-sense RNA virus belonging to the genus Enterovirus of the picornavirus family [1–4]. EV-A71 is known for its high infectivity and its ability to mutate rapidly and cause serious illness. Pathological conditions caused by EV-A71 infection include hand-foot-mouth disease, herpes virus, and neurological complications [5, 6]. However, a detailed understanding of the pathogenesis of EV-A71 is lacking. To date, there are no specific vaccines, antiviral drugs or therapies for EV-A71 infection. Therefore, further research is needed to analyze the underly mechanism of the interaction between EV-A71 and host cell factors.

MicroRNAs (miRNAs) are a group of the short non-coding ribonucleic acid molecules (approximately 22 nucleotides) whose function to regulate the activity of specific mRNA targets [7, 8]. miRNAs target specific cellular mRNA by base-pairing with complementary sequences in the mRNA and inhibiting protein expression, thereby regulating cellular processes such as cell growth, proliferation, differentiation, and apoptosis [9–11]. In addition, some studies have reported that many specific miRNAs play important roles in viral replication [12]. Some viruses regulate viral genes expression through host miRNAs and virus-encoded miRNAs. For example, HCV depends on miR-122 expression, which interacts with the 5’UTR of HCV RNA and then increases HCV replication in hepatocytes [13, 14]. In contrast, West Nile virus encodes its own viral miRNA, which acts like a cellular miRNA and enhances WNV replication in mosquito cells [15]. Although many studies have reported that EV-A71 replication is regulated by miRNAs, such as miR-141 [16] and miR-548 [17], and that the infection and pathogenesis of EV-A71 infection will also be regulated by miRNAs. Unfortunately, miRNA involvement in EV-A71 infection or replication remains unknown.

The cyclic AMP-competitive element-binding protein 5 (CREB5) functions as a protein-coding gene in eukaryotic cells and reported to be a transcription factor [18, 19]. Some studies report the development of the cancers was regulated by CREB5. For example, when CREB5 was overexpressed, it up-regulates CSF1R, MMP9, PDGFRB, FIGF, and IL6 to promote metastasis of colorectal cancer [20]. In addition, when CREB5 expression is higher in patients with the epithelial ovarian cancer, their survival rate and the no-recurrence rates are low [21]. Some studies have shown that CREB5 is a negative regulator of viral infection. For example, overexpressing the CREB5 reduced HBV replication and HBV protein production in Huh7 cells [22]. Another study showed that CREB5 is involved in the cellular bioregulatory module of the frontal cortex, a function that is disrupted in HIV encephalitis [23].

In our previous study, we analyzed differentially expressed miRNAs from the sera of patients infected with EV-A71. Among upregulated circulating miRNAs, miR-876-5p has been identified to be closely related to severe symptoms of the disease [24]. In the present study, we demonstrated that miR-876-5p is upregulated in EV-A71-infected neuroblastoma cells. Overexpression of miR-876-5p or knockdown of CREB5 promoted the EV-A71 viral replication, while downregulation of miR-876-5p or overexpression of CREB5 resulted in inhibition of EV-A71 replication and viral protein expression. In addition, miR-876-5p was shown to directly target CREB5. Furthermore, CREB5 silencing reduced the antiviral effect of anti-miR-876-5p treatment on EV-A71 replication and viral protein expression. These results indicate that miR-876-5p promotes the EV-A71 infection cycle by targeting the CREB5 protein, thus providing a new antiviral strategy for EV-A71 infection. The results of the aforementioned studies suggest a new approach to knock out essential cellular miRNAs as an antiviral strategy.

## Methods

### Enteroviruses and cell line

The human neuroblastoma, SF268, cells were cultured in the DMEM medium (Gibco-BRL, Carlsbad, CA, USA) without or with 10% FBS + pencillin/stretomycon (Gibco-BRL, Carlsbad, CA, USA) in the incubator supplemented with atmosphere of 5% CO_2_. *Rhabdomyosarcoma cells (RD cells)* cells were used to amplify the EV-A71 (Taiwan strain 2231), and the viral titers were determined by plaque assay. To carry out the viral infection, SF268 cells were first incubated in serum free medium for 2 h prior to infection. Cells were incubation with EV-A71 for 2 h at 37 °C. The viral-infected cells were then washed with PBS followed by culturivation in the complete medium as described above at the indicated time points before harvest.

### Construction of the flag-tagged CREB5 plasmids

All primer sequences are written in the 5′ to 3′ orientation. To generate the 3xFlag-CREB5 plasmid, we PCR amplified the full-length of CREB5 from the RNA using the forward primer, GCTTATGACCGGGATGCCTGAGGAAGTGCACC, and the reverse primer, GCTCTTTACCCGACTTCTTCCATGCG. The correct PCR product was then purified and digested with *Eco*RI and *Xho*I, and was ligated into the pCMV-8-Flag vector.

### Plaque assay

Virus quantification was carried out by plaque forming assay. The procedures for plaque assay involves counting the number of viruses have been described elsewhere [24]. In brief, SF268 cells were cultured in an 6-well plate at 4 × 10^5^ cells and incubation overnight to form a monolayer. EV-A71 viral particles were prepared and 10-fold serial dilutions in DMEM (serum-free) medium prior to viral infection. SF268 cells were infected with viral particles for 2 h to allow the occurrence of infection. After incubation, the cells were washed with PBS and added 2 ml of 0.3% agarose (Invitrogen) to each well and then incubation at room temperature to solidify the agarose. The cells were then cultured at 37 °C for 4 additional days before fixed with 10% formaldehyde. The fixed cells were stained with a solution of crystal violet, formalin, ethanol, and NaCl (Sigma), respectively for 2 min. Virus titers were calculated as the number of plaques X and is expressed as PFU/ml.

### Western blotting and antibodies

We performed SDS-PAGE and western blotting to detect the CREB5 and viral proteins. Total protein sample isolation and separation have been described elsewhere [25]. In brief, the cellular total proteins was boiled and separation in an 12% SDS-PAGE. The separated SDS-PAGE was then transferred to a polyvinylidene difluoride (PVDF) membrane (Bio-Rad Laboratories, Hercules, CA) and then washed. The membrane was and blocking with fast blocking solution (Biofuture Co., Taoyuan, Taipei) for 3 min prior to the primary and secondary antibodies incubation. The primary antibodies used were listed as the following: rabbit anti-EV-A71 VP (GeneTex, 1:1000), anti-Flag (Sigma, 1:3000 dilution) and anti-EV-A71 3D (1:5000 dilution); for detection of CREB5 (GeneTex, 1:2000) and β-actin (Sigma, 1:10000). The secondary antibodies were purchased from – (a) Goat anti-rabbit HRP secondary antibody (Abcam ab205718); (b) Goat anti-rabbit HRP secondary antibody (Abcam ab205719). The primary and secondary antibodies were diluted in the Super antibody mate solution (MDBio Co., Ltd., Taipei, Taiwan) followed by incubation ECL (BioMan biotech., Taipei, Taiwan) with the membranes. The signals of interested were detected using the ImageQuant™ LAS 4000 biomolecular imager (GE, USA).

### Knockdown of CREB5

The siRNA specific for CREB5, 5′ CAGAAGUUAAGGAGAAUUA 3′, was synthesized by Sigma. To knockdown the CREB5, SF268 cells were treated with siCREB5 or scamble siRNA into cells using RNAiMAX lipofectamine (Invitrogen) in Opti-MEM reduced serum medium (Invitrogen). The transfection of siRNA have been described elsewhere [25], and CREB5 expression was downregulated after 24-h silence.

### Mice experiments

For mice experiments, the animal care and experimental procedures are adhered to the protocol approved by the Institutional Animal Care and Use Committee of Chang Gung University (IACUC approval no.: CGU-14-113). The pathogen-free ICR mice (7-day-old) were injected (intraperitoneally) in 50 μL of PBS-dissolved miRNA hairpin inhibitor (NC, purchased from Dharmacon, Lafayette, CO, USA) or PBS-dissolved anti-miR876-5p hairpin inhibitor (Dharmacon, Lafayette, CO, USA) with the dose of 25 mg/Kg using the 30G syringe. The treated mice are then inoculated with 10^6^ PFU of EV-A71 mp4 virus (with a total volume of 50 μL) using the same syringe.

### RNA preparation, reverse transcription, and quantitative real-time PCR

We used TRIzol (Invitrogen) solution to isolate total RNA in accord to the manufacture’s protocol. To determine the mRNA level for CREB5, we first produce the cDNA by reverse transcription (ABI) using 3 μg of total RNA as the template. The primers we used are as the following:

(a) EV71 Forward primer: 5′-TCAATTCCCGTTTCTCATCCA-3′; Reverse primer: 5′-GAGGGAGCGCACGTGATCT-3′ (b) CREB5 Forward primer: 5′-ATCACCAGACCTCGCCACAT-3′; Reverse primer: 5′ GCTGGGGTGGCTGTATTGTC 3′ (c) miR-876-5p Forward primer: 5′-TGAAGTGCTGTGGATTTCTTTGTG-3′; Reverse primer: 5′ -TGAATTACTTTGTAAACCACCACCA-3.

The quantitative real time PCR (qPCR) assay was used to detect the CREB5 and the EV-A71 cDNAs. The qPCR analyses were performed in duplicate using SYBR green master mix (Bio-Helix, Keelung, Taiwan) in StepOne qPCR system (ABI). The beta-actin as a cellular total RNA expression (beta-actin is a house-keeping expressed gene) to quantify EV71 RNA, CREB5 RNA and miR-876-5p levels.

### Construction of luciferase reporter plasmids

The luciferase reporter plasmids were constructed based on pmiR-report™ (Invitrogen). Briefly, CREB5/CREB5-mut. (seed region 2–4 nt; mutation), EV71–5’UTR/EV71–5’UTR-mut. (seed region 2–4 nt; mutation) were amplified by PCR and then cloned into the pmiR-REPORT™ (Invitrogen) plasmid by SpeI and HindIII restriction sites. Normalization for the DualLuciferase® Reporter Assay System (Promega, Madison, WI, USA) bases on the pRL-TK plasmid that expresses the Renilla luciferase gene at low levels from a minimal herpes simplex thymidine kinase promoter.

### Statistical analysis

All the data were presented as means ± standard deviation except means ± standard errors. All variables were tested for normal distributions using the Kolmogorov-Smirnov test. The Student’s *t-*test was applied to compare the means of continuous variables and normally distributed data; otherwise, the Mann-Whitney *U* test was employed. The difference of the categorical variances was analyzed by Pearson’s chi square test or Fisher’s exact test. A value of *p* < 0.05 was considered to represent a significant difference.

## Results

### miR-876-5p promoted EV-A71 replication and expression

**Fig. 1:**
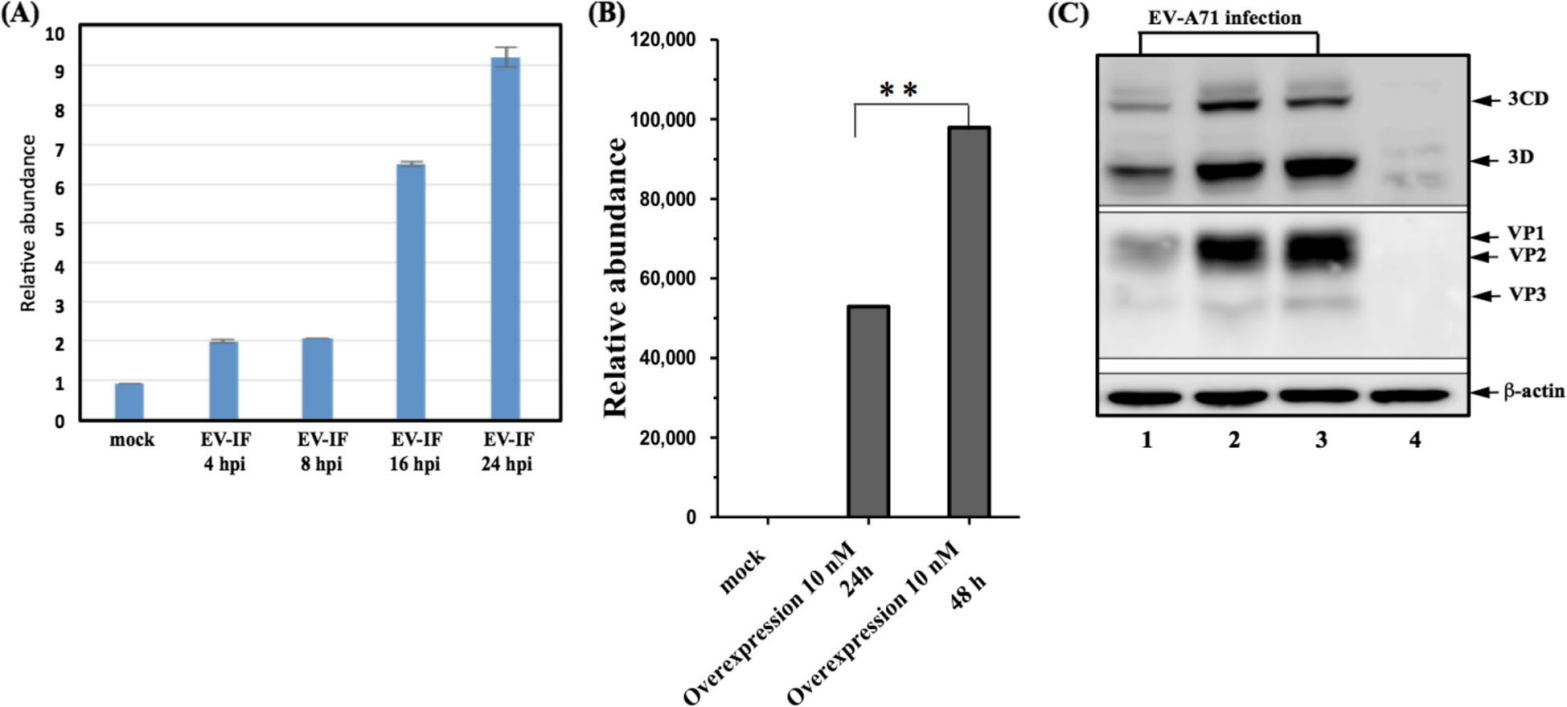
miR-876-5p facilitated the EV-A71 infection cycle in human neuroblastoma SF268 cells. **a** SF268 cells were infected with EV-A71 at an MOI of 1, and the cells were harvested at different time points after infection, as indicated. The RNA was the isolation and expression level of miR-876-5p, which was determined through real-time quantitative polymerase chain reaction (RT-qPCR); **b** The expression level of miR-876-5p was detected through RT-qPCR after in the pmiR-876-5p transfected cells; **c** SF268 cells were expressed with miR-876-5p (lanes 2–3), followed by infection with EV-A71. The intracellular EV-A71 viral proteins, 3D/3CD, and VP1–3 were detected through Western blotting (lane 1: control cells; lanes 2–3: miR-876-5p overexpressed; lane 4: mock-infected cells). β-actin was loaded as the internal control. All experiments were repeated three times. ** *P* < .01

**Fig. 2:**
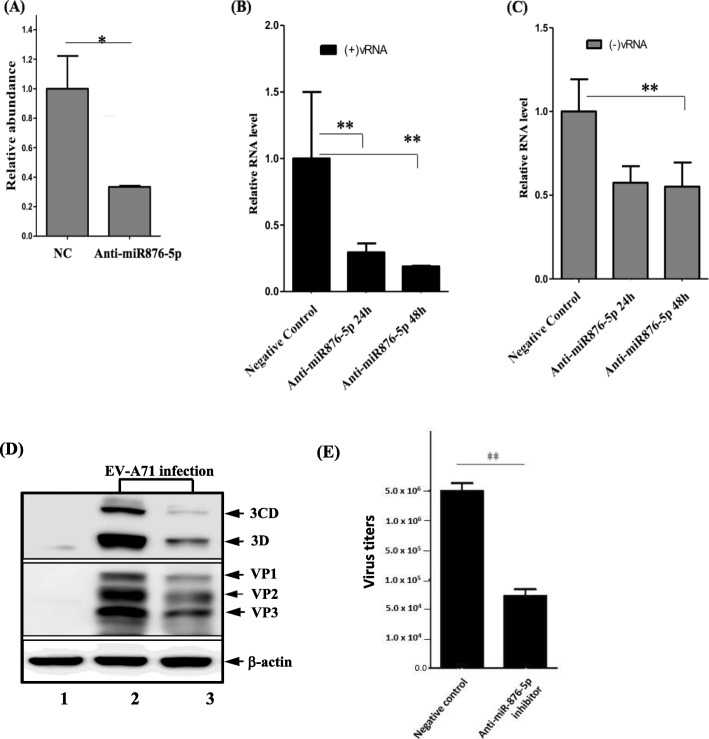
miR-876-5p preferentially regulated the EV-A71 life cycle. **a** SF268 cells were introduced to an anti-miR-876-5p inhibitor and then cultivated for 24 h. The expression level of miR-876-5p was quantified by real-time polymerase chain reaction (RT-qPCR). The anti-miR-876-5p-inhibitor-treated cells were infected with EV-A71 (MOI = 1) for 24 h. Both the (+)strand of viral RNA (**b**) and (−)strand of viral RNA (**c**) were quantified through RT-qPCR; **d** protein expression levels of viral 3D/3CD and VP1–3 were detected by Western blot using anti-3D and anti-VP specific antibodies (lane 2: Negative control cells; lane 3: anti-miR-876-5p inhibitor treatment); **e** The infectivity of EV-A71 progeny from the anti-miR-876-5p-inhibitor-treated cells was assessed in the plaque assay. The virus titers are shown in PFU/mL. The data shown are based on the mean of three independent experiments. * *P* < .05; ** *P* < .01

In order to explore the role of miR-876-5p in the EV-A71 infection cycle, the expression of miR-876-5p in SF268, a human neuroblastoma cell line, was initially evaluated at different time points by real-time quantitative polymerase chain reaction after EV-A71 infection. Compared to mock control cells, a significant increase in miR-876-5p was observed immediately at 2 h postinfection and a 9-fold increase over mock-infected cells at 24 h after EV-A71 infection (Fig. 1a). Subsequently, the miR-876-5p plasmid was transfected into SF268 cells for gain-of-function experiments. The overexpression effects of miR-876-5p were detected in the cells transfected with miR-876-5p plasmids (at the concentration of 10 nM) at 24 h and 48 h, respectively (Fig. 1b). Then, the effect of miR-876-5p on the expression of EV-A71 viral proteins was investigated by measuring the expression level of viral protein. Compared with the miR control group, the viral 3D/3CD proteins and VP1/VP3 proteins were dramatically increased in SF268 cells (Fig. 1c, lanes 2–3). However, treatment with anti-miR-876-5p inhibitor for 24 h resulted in lower expression of miR-876-5p than control cells (Fig. 2a). It is estimated that anti-miR-876-5p inhibits EV-A71 viral RNA replication and RNA replicative intermediates (negative-stranded RNA) in SF268 cells (Fig. 2b & c).Western blot analysis revealed that viral 3D/3CD proteins and VP1–3 proteins were dramatically reduced in anti-miR-876-5p-transfected cells (Fig. 2d, lane 3). Furthermore, a decrease in the yield of progeny virus was also observed by performing the plaque-forming assay as shown in Fig. 2e. Overall, our results suggest that miR-876-5p promotes replication and expression of EV-A71 in human neuroblastoma SF268 cells.

### miR-876-5p targets CREB5 protein during EV-A71 infection

**Fig. 3:**
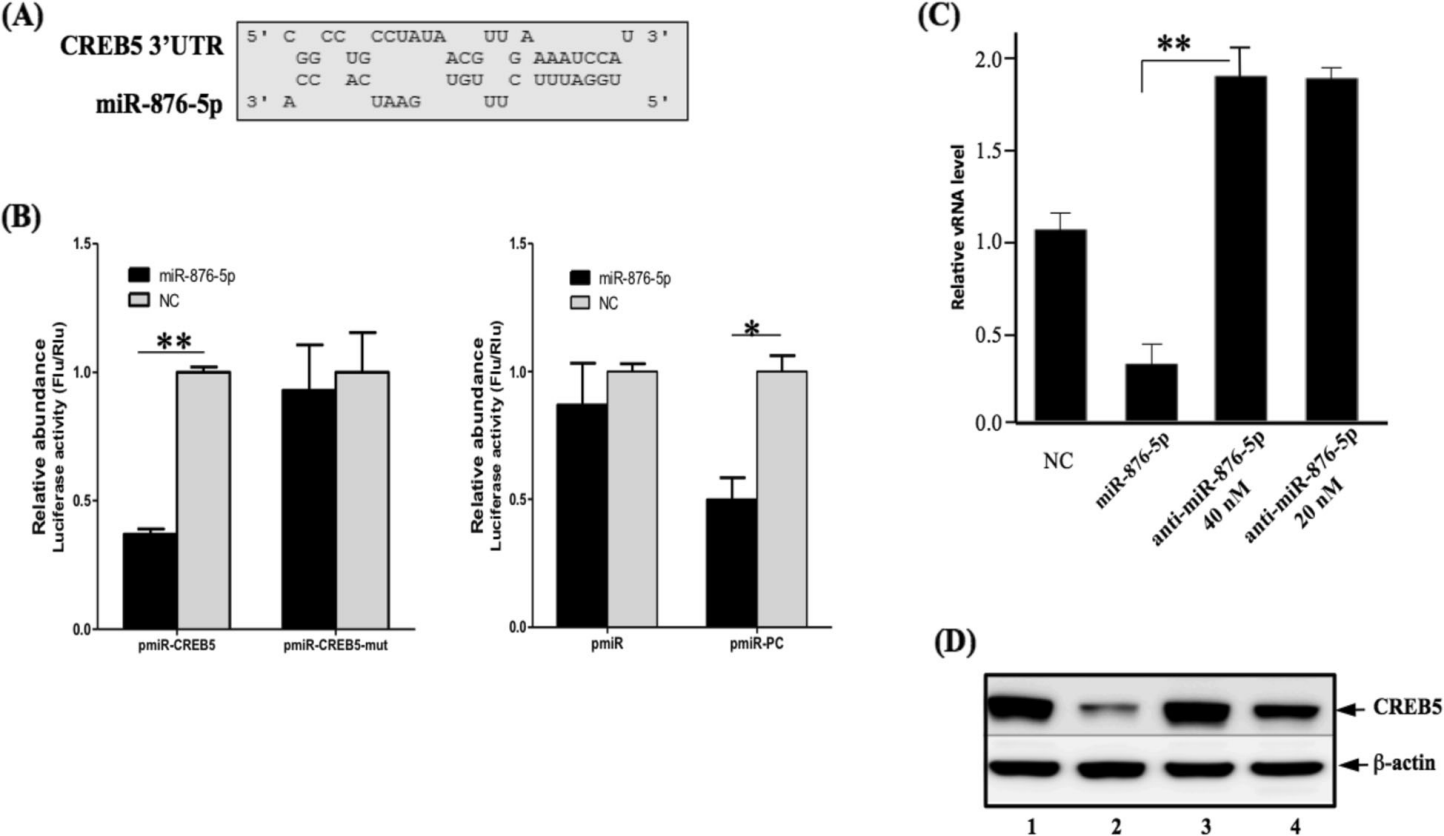
CREB5 was a direct-targeted gene of miR-876-5p. **a** Predicted binding sites between miR-876-5p and 3’UTR of CREB5. **b** Luciferase activities of SF268 cells were determined through the luciferase reporter assay by cotransfection with CREB5–3’UTR or CREB5–3’UTR-mutant and miR-876-5p or miR-control. The expression level of CREB5 at mRNA (**c**) and protein (**d**) levels was measured through real-time quantitative polymerase chain reaction and Western blotting in SF268 cells transfected with miR-876-5p (lane 2), anti-miR-876-5p inhibitor (20 and 40 nM; lanes 3 and 4), or corresponding controls (lane 1). All experiments were repeated three times. * *P* < .05; ** *P* < .01

To explore the potential endogenous targets of miR-876-5p involved in the EV-A71 infection cycle, we adopted a global view of differentiated expressions of host gene profiles that respond to EV-A71 infection (Additional file 1: Table S1). The cDNA array was performed to determine the effect of changes in expressed host genes on EV-A71 infection. Based on cDNA array analysis, we obtained five significant reduced mRNA expressions for EV-A71 infection (Table 1). Secondly, by searching the miRNA target genes in TargetScan and MiRDB, we found that miR-876-5p contains complementary binding sites in the 3’UTR of CREB5 (Fig. 3a), which is consistent with our microarray result, indicating that CREB5 is significantly downregulated host proteins in response to EV-A71 infection. Next, luciferase reporter plasmids (pmiR-CREB5 and pmiR-CREB5 mut) was established, which contained a wild-type or one mismatch miR-876-5p binding sites (mutated) in 3’UTR of CREB5 and cotransfected with miR-control or miR-876-5p into SF268 cells. Detection of the luciferase reporter gene revealed that cotransfection of miR-876-5p and pmiR-CREB5 (overexpression) with the control group significantly impeded luciferase activity compared to the control group (Fig. 3b), whereas cotransfection with miR-876-5p and pmiR-CREB5 mutations led to less changes in luciferase activity. Next, we observed a significant reduction of CREB5 expression at mRNA (Fig. 3c) and protein levels in the miR-876-5p overexpressed cells (Fig. 3d, lane 2). It is worth noting that similar CREB5 protein expression levels were detected in anti-miR-876-5p inhibitor-treated cells (Fig. 3d, lanes 3–4), suggesting that miR-876-5p suppressed the expression of CREB5 by targeting its 3’UTR.

**Table 1:** The down-regulated proteins identified by cDNA array from mock-infected and EV71-infected human blastoma SF268 cells

No.	NCBI number	Gene Name	Protein Name	Fold- change
1	NM_182898	CREB5	cAMP responsive element binding protein 5	−2.695
2	NM_203349	SHC4	Src homology 2 domain containing family, member 4	−2.488
3	NM_001204375	NPR3	natriuretic peptide receptor C/guanylate cyclase C	−2.719
4	NM_025184	EFHC2	EF-hand domain (C-terminal) containing 2	−2.448
5	NM_020801	ARRDC3	arrestin domain containing 3	−2.208

### CREB5 inhibited EV-A71 replication and expression

**Fig. 4:**
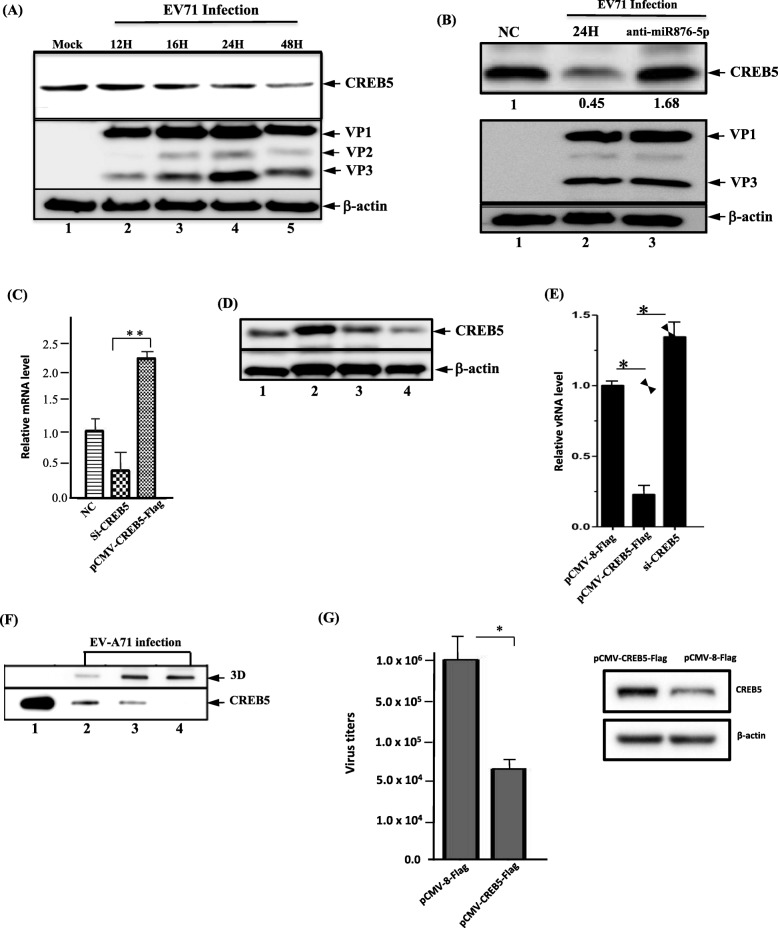
CREB5 repressed EV-A71 viral replication and expression. **a** SF268 cells were mock-infected or infected with EV-A71 (MOI = 1) for different time points as indicated (lanes 2–5. 12–48 h after infection). Cell lysates were then harvested for detection of CREB5 protein levels by Western blotting using the anti-CREB5 specific antibody. **b** Mice were treated with the miRNA hairpin inhibitor negative control in phosphate buffered saline (lane 1) or anti-miR-876-5p inhibitor for 24 h and infected with EV-A71. The protein extracted from the site of injection in the abdominal muscle was collected for CREB5 and viral protein investigations; VP1–3 analysis was performed with Western blotting using specific antibodies as indicated. Lane 1, the miRNA hairpin negative control and no EV-A71 infection; lane 2, the miRNA negative control of mice infected with EV-A71 or anti-miR876-5p inhibitor-treated (lane 3) mice. **c** Quantitative polymerase chain reaction (qPCR) analysis of CREB5 mRNA level in si-CREB5-transfected or CREB5-overexpressed cells. **d** Expression of CREB5 determined by Western blotting in pCMV-CREB5-Flag-transfected cells (lane 2) or siCREB5-transfected cells (lanes 3–4). Lane 1, cells were transfected with pCMV-8-Flag plasmid as the negative control. **e** Accumulation of viral RNA in pCMV-CREB5-Flag-transfected cells or siCREB5-transfected cells upon EV-A71 infection in SF268 cells. pCMV-8-Flag is the empty plasmid used for transfection control. **f** Expression level of viral protein and 3D and CREB5 protein in the mock-infected, pCMV-CREB5-transfected, and siCREB5-transfected cells upon EV-A71 infection. Lane 1, Mock-infected cells used as negative control; lane 2, cells were treated with si-scramble RNA and infected with EV-A71 for 24 h; lanes 3–4, cells were treated with siCREB5 RNA (5 ng and 10 ng) and infected with EV-A71 for 24 h. **g** Viral titers of pCMV-CREB5-Flag-transfected or pCMV-8-Flag control and EV-A71 infected cells. All experiments were repeated three times. * *P* < .05; ** *P* < .01

To confirm the function of CREB5 in the EV-A71 infection cycle, SF268 cells were infected with EV-A71 at different time points (12, 16, 24, and 48 h), and then cell lysates were harvested to evaluate CREB5 protein levels. As shown in Fig. 4a, the CREB5 protein were significantly reduced in EV-A71-infected cells compared with mock-infected cells. In addition, our laboratory reported that CREB5 protein was also increased in mice treated with anti-miR876-5p-inhibitor(Fig. 4b). Next, the SF268 cells were transfected with pCMV-8-Flag, pCMV-8-CREB5-Flag, si-scramble, or si-CREB5. First, compared to control cells, the mRNA of CREB5 showed a significantly increased level of cells transfected with pCMV-8-CREB5-Flag (Fig. 4c). Similarly, CREB5 protein was notably enhanced in cells transfected with pCMV-8-CREB5-Flag (Fig. 4d, lane 2), and significantly down-regulated in si-CREB5-transfected cells (Fig. 4D, lanes 3–4). Upon EV-A71 infection, we found that EV-A71 viral RNA accumulation was significantly reduced (Fig. 4e), and viral protein production was reduced in CREB5-overexpressed SF268 cells. In contrast, accumulation of viral RNA levels and increased levels of expressed viral protein levels were confirmed in the siCREB5-transfected cells (Fig. 4f). In addition, the plaque-forming assay suggest that progeny viruses levels are significantly reduced in CREB5 overexpressed cells (Fig. 4g) and significantly elevated in si-CREB5-transfected cells. All the above data indicate that CREB5 protein plays an inhibitory role in the EV-A71 infection cycle.

### miR-876-5p modulates EV-A71 replication and expression through targeting on CREB5

**Fig. 5:**
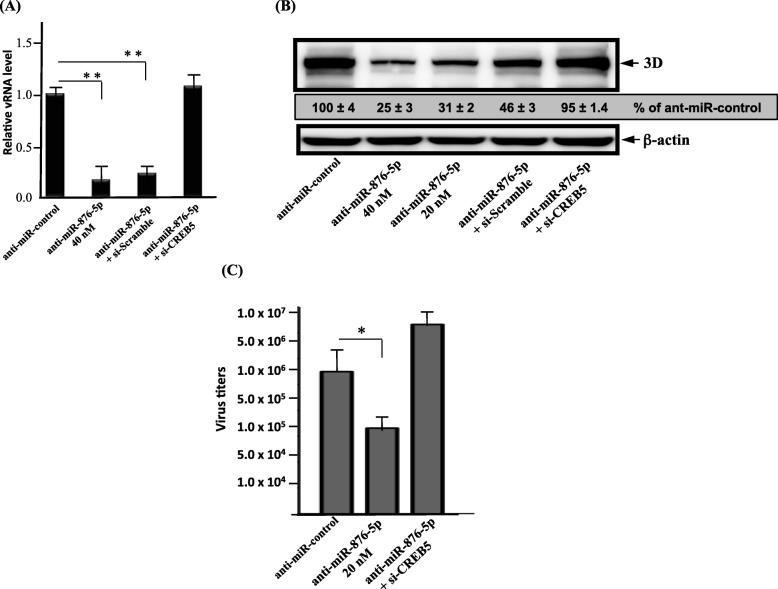
Downregulation of miR-876-5p inhibited EV-A71 replication and expression by modulating CREB5. SF268 cells were transfected with either anti-miR-876-5p-inhibitor only or in combination with si-CREB5 or si-Scramble as negative control. **a** SF268 cells were treated with the miRNA hairpin negative control (anti-miR-control), anti-miR-876-5p, anti-miR-876-5p and si-scramble RNA (the negative control for si-CREB5 RNA), or anti-miR-876-5p and si-CREB5 RNA for 24 h, followed by EV-A71 infection. The cells were harvested at 24 h after the EV-A71 infection, and viral RNA was examined by real-time-qPCR of each sample. **b** EV-A71 viral 3D protein was detected by Western blotting using anti-3D specific antibody. β-actin was detected as the loading control. **c** Progeny viruses were measured in culture supernatant by a plaque-forming assay. All experiments were repeated three times. * *P* < .05; ** *P* < .01

Next, we evaluated whether the role of miR-876-5p in the EV-A71 infection cycle is mediated by CREB5. Overexpression experiments were performed in SF268 cells by transfection with an anti-miR-876-5P inhibitor or in combination with an si-CREB5 plasmid. As expected, the CREB5 knockdown apparently abolished anti-miR-876-5p-induced reduction in viral RNA accumulation (Fig. 5a) and production of various viral proteins (Fig. 5b). In addition, the down-regulation of miR-876-5p significantly reduced viral infectivity (Fig. 5c), which was reversed by the knocked down CREB5 expression level. Overall, these results demonstrate that miR-876-5p can regulate the EV-A71 infection cycleby targeting the CREB5 host factor.

## Discussion

Host response is an important clue to determine the pathogenesis of EV-A71 infection. We have characterized serum circulating miRNA profiles of various EV-A71-infected diseases [24]. Compared with healthy controls, after EV-A71 infection, a total of 44 miRNAs were found to increase at least two-fold, while EV-A71 infected patients had a reduction of 133 miRNAs. The elevated expression of miR-876-5p can also be detected in mice, and further has been performed because knockdown of miR-876-5p can reduce the severity of symptoms caused by EV-A71 infection in mice [24]. Our past studies have confirmed that circulating miR876-5p is a response in patients with severe EV-A71 infection. However, no molecular evidence has been sought. In the present study, we provided evidence that miR-876-5p participates in the EV-A71 infection cycle through targeting CREB5 in SF268 cells, opening up a new application tool for circulating miRNAs in the EV-A71 infection.

Many reports indicate that intracellular miRNAs are involved in the EV-A71 infection process and the pathogenesis caused by EV-A71 [26–29]. MicroRNAs are referred to as small, single-stranded, and non-coding RNAs. The main function of miRNAs is to mediate post-transcriptional silencing of target genes. In general, miRNAs bind to specific sites in the 3′ UTR of the target gene and degrade cellular mRNA, thereby regulate cell homeostasis [30]. The main role of miRNAs is to inhibit protein expression and induce mRNA degradation [31]. There have been many reports of miRNAs as essential regulators of biological processes, including immune responses, development, and cancer [32]. It is generally believed that many specific miRNAs are involved in regulating crosstalk between the host and the viral-infection-causing pathogens, and play an important role in host response to the viral infections [33]. Our results indicate that miR-876-5p regulates viral replication by targeting the CREB5 protein. In deed, the regulation of miRNAs with specific genes in cells can modulate specific biological pathways. The extensive dysregulation of miRNA expression may reflect multiple cellular defense responses caused by inflammation, bacterial infections, and viral infections [34–36]. Upon viral infection, the host can alter several specific biological pathways, such as differentiated growth, development, apoptosis, changes in metabolic pathway, and homeostasis. In recent years, there has been increasing evidence that miRNAs participate in specific signaling pathways through the regulation of multiple functionally related proteins, which are involved in the pathways [37, 38]. Our results show that the regulation of miRNAs in the viral infection cycle can be demonstrated by targeting at least one host factor involved in viral replication or expression.

There is increasing evidence that some small RNA molecules are involved in the EV-A71 infection cycle. For instance, upregulated expression of circulating miR876-5p is a response to severe EV-A71 infection. After viral infection, host miRNAs can be used to enhance immune system process. It has been confirmed that miRNA can increase (e.g., miRNA-146a and miRNA-548) or inhibit (e.g., miRNA-526a) survival during EV-A71 infection in conjunction with a related host molecule [39, 40]. In addition, other studies have confirmed that host cellular miRNAs in Hep2 cells and RD cells show significant changes in response to EV-A71 infection, suggesting that altered miRNA expression plays an important role in the interaction of EV-A71 withhost [41]. Nevertheless, the regulation of specific miRNA expression levels in response to EV-A71 infection is still not fully understood. Subsequently, in order to further elucidate the key points of the differential miRNA in the host after EV-A71 infection, many studies have attempted to isolate a significant number of differentially expressed miRNAs and identified opposite expression trends. For example, miRNA-1246 has been found to be one of p53’s target genes in the carcinogenesis of many different cancers, including cervical cancer, esophageal cancer, colorectal cancer, pancreatic cancer, and liver cancer [42]. Some reports indicatethat different miRNA profiles of EV-A71 infection were obtained from various infected cells or clinical collections. For example, several miRNA profiles obtained from the peripheral blood mononuclear cells of EV-A71-infected rhesus monkeys identified several miRNAs known to have differential expression involved in cellular immune processes. These are important miRNAs and they are the basis for various immune responses caused by EV-A71 infection [26]. In addition, a study identified miR-143, miR-324-3p, and miR-545 as key miRNAs that distinguish EV-A71 infection in HFMD patients and provide complementary biomarkers for the diagnosis and classification of enterovirus HFMD infection [41]. Overall, host miRNAs act as key effector molecules in the EV-A71 infection cycle.

As mentioned above, some advanced technologies and bioinformatics methods have been used to identify differentially expressed miRNAsas a response to EV-A71 infection in cells. Although these studies highlight the specific differential expression patterns of miRNAs in response to EV-A71 infection, the exact function of these miRNAs in the pathogenesis of the virus remains to be determined. Past research has used bioinformatics methods to screen differentially expressed miRNAs from trend analysis to predict target GO. Pathway analysis provides comprehensive information about the differential biological functions and pathways of EV-A71 infection. In addition, the construction of a detailed regulatory networks can greatly help to understand the miRNA-mediated mechanisms associated with EV-A71 infection. However, these results are limited to functional predictions, and most data do not reflect true physiological function in the responding cells. Therefore, this study directly used cell lines to verify that miRNA target gene overexpression or gene silencing to explore its role in EV-A71 infection. Experimental results yielded host–pathogen interactions and pathogenesis, as well as more insights into the mechanism.

## Conclusions

Host miRNAs are known to function as key effector molecules in the RNA virus infection cycle. In this study, we provide evidence that miR-876-5p participates in the EV-A71 infection cycle by targeting CREB5 in SF268 cells, and we demonstrate that miR-876-5p regulates viral replication by targeting CREB5 protein because CREB5 protein plays an inhibitory role in the EV-A71 infection cycle. This study opens new tools for circulating miRNAs in EV-A71 infection.

## Supplementary information

**Additional file 1: Table S1.** Microarray data of the differentially expressed genes obtained from mock-infected (M), EV-A71 infected wild type cells (24 hpi) and EV-A71 infected with miR876-5p knocked-down (KD infection) cells.

## Data Availability

All data generated or analyzed during this study are included in this published article.

## Abbreviations

5’UTR: Untranslated region
CREB5: Cyclic-AMP responsive element binding protein 5
EV-A71: Enterovirus A71
HCV: Hepatitis C virus
HFMD: Hand-Foot and Mouth disease
PFU: Plaque forming unit
