# Supplementary material for: MicroRNA 876-5p modulates EV-A71 replication through downregulation of host antiviral factors
Source: Virol J. 2020 Feb 5;17:21. doi: 10.1186/s12985-020-1284-8 (PMC7003331; doi:10.1186/s12985-020-1284-8)
Supplement: Supplementary file 1 — Additional file 1: Table S1. Microarray data of the differentially expressed genes obtained from mock-infected (M), EV-A71 infected wild type cells (24 hpi) and EV-A71 infected with miR876-5p knocked-down (KD infection) cells. [file 12985_2020_1284_MOESM1_ESM.zip › supplementary material legend.docx]

Supplementary material

**Table S1.** Microarray data of the differentially expressed genes obtained from mock-infected (M), EV-A71 infected wild type cells (24 hpi) and EV-A71 infected with miR876-5p knocked-down (KD infection) cells.
